# Pharmacological Effects of Resveratrol in Intervertebral Disc Degeneration: A Literature Review

**DOI:** 10.1111/os.13560

**Published:** 2022-10-27

**Authors:** Ming‐yang Liu, Liang Zhang, Wei‐dong Zang, Kai‐guang Zhang, Hai‐jun Li, Yan‐zheng Gao

**Affiliations:** ^1^ Present address: Henan Province Intelligent Orthopedic Technology Innovation and Transformation International Joint Laboratory, Henan Key Laboratory for Intelligent Precision Orthopedics, Department of Surgery of Spine and Spinal Cord, Henan Provincial People's Hospital People's Hospital of Zhengzhou University, People's Hospital of Henan University Zhengzhou China; ^2^ Department of Human Anatomy, School of Basic Medical Sciences Zhengzhou University Zhengzhou China; ^3^ Department of Immunity, Institute of Translational Medicine The First Hospital of Jilin University Jilin China

**Keywords:** Apoptosis, Autophagy, Extracellular matrix degradation, Intervertebral disc degeneration, Resveratrol, Senescence

## Abstract

Intervertebral disc degeneration (IDD) is a high incidence disease of musculoskeletal system that often leads to stenosis, instability, pain and even deformity of the spinal segments. IDD is an important cause of discogenic lower back pain and often leads to large economic burden to families and society. Currently, the treatment of IDD is aimed at alleviating symptoms rather than blocking or reversing pathological progression of the damaged intervertebral disc*.* Resveratrol (RSV) is a polyphenol phytoalexin first extracted from the *Veratrum grandiflflorum O. Loes* and can be found in various plants and red wine. Owing to the in‐depth study of pharmacological mechanisms, the therapeutic potential of RSV in various diseases such as osteoarthritis, neurodegenerative diseases, cardiovascular diseases and diabetes have attracted the attention of many researchers. RSV has anti‐apoptotic, anti‐senescent, anti‐inflammatory, anti‐oxidative, and anabolic activities, which can prevent further degeneration of intervertebral disc cells and enhance their regeneration. With high safety and various biological functions, RSV might be a promising candidate for the treatment of IDD. This review summarizes the biological functions of RSV in the treatment of IDD and to facilitate further research.

## Graphical abstract

The intervertebral disc acts as the load‐bearing component of the spine and is composed of three closely connected parts: inner nucleus pulposus (NP), peripheral annulus fibrosus (AF) and outer cartilage endplate (CEP). IDD is a complex disease including multiple pathological processes. The pathogenesis of IDD mainly involves degradation of ECM, NP cells senescence, apoptosis, autophagy, inflammatory responses and oxidative stress. NP cells senescence, apoptosis, inflammation and oxidative stress result in the promotion of the ECM degradation. What is more, activation of autophagy can also influence the activity of NP cells, thereby regulating ECM homeostasis. According to the literatures, the RSV showed protective effects on IDD through a variety of mechanisms.

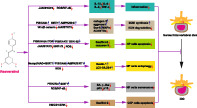

## Introduction

Intervertebral disc degeneration (IDD) is a high incidence disease of musculoskeletal system that often leads to stenosis, instability, pain and even deformity of the spinal segments.^1^, ^2^, ^3^ Many IDD patients lose their ability to work, thereby imposing a large economic burden to their families and society.^4^, ^5^, ^6^, ^7^ Current treatments mainly focus on alleviating the symptoms rather than limiting or reversing pathological changes of disc degeneration, which means the treatment outcomes and prognosis are far from ideal.^8^, ^9^ The pathogenesis of IDD is related to nucleus pulposus (NP) cells apoptosis, NP cells senescence, inflammation, oxidative stress, and extracellular matrix (ECM) degradation. Therefore, suppressing the inflammatory response and oxidative stress, inhibiting NP cells senescence and apoptosis and promoting the biosynthesis of the ECM are pivotal to IDD treatment.^10^, ^11^

Resveratrol (RSV, C_14_H_12_O_3_) is a polyphenol phytoalexin with a relative molecular weight of 228. It was first extracted from *Veratrum grandiflorum* O. Loes by Takaoka in 1939 and can be found in various plants and red wine.^12^ It has both cis‐ and trans‐isomers when found in nature, of which the trans‐isomer provides the main biological benefits due to the lower steric hindrance of its side chains.^13^, ^14^ Modern pharmacological studies show that RSV has protective effects against various diseases, such as osteoarthritis, neurodegenerative diseases, cardiovascular diseases, and diabetes.^15^, ^16^, ^17^, ^18^ The therapeutic effects of RSV on IDD are related to its antioxidant and anti‐inflammatory activities.^19^, ^20^, ^21^ Moreover, RSV also affects NP cells apoptosis, autophagy, and ECM biosynthesis through multiple signal transduction pathways.^19^, ^20^, ^22^, ^23^ At present, researches of RSV on the treatment of IDD are limited to preclinical studies. This review summarizes the biological functions of RSV in IDD treatment. The limitations of current studies and new insights for further studies are also provided at the end of article.

## Pharmacokinetics and Toxicity

In 40 healthy volunteers orally administered with single doses of 0.5, 1, 2.5, or 5 g RSV, the mean maximum plasma concentration (Cmax) at the highest dose was 539 ng/mL, which was achieved within 1.5 h post dose (Tmax).^24^ The elimination half‐life of RSV was 8.52 h, while the total exposure (AUC_0–∞_) was 1319 ng h/mL.^24^ The distribution research in animal models indicates that the highest tissue concentration of RSV was found in the brain, liver, intestine, and fat.^25^ After absorption through passive diffusion or carrier‐mediated processes, RSV is mainly metabolized in enterocytes and hepatocytes.^26^, ^27^ It can be rapidly metabolized into RSV‐glucuronides and RSV‐sulfates *via* uridine‐5′‐diphosphate‐glucuronosyltransferase and sulfotransferase, respectively.^28^, ^29^ In addition, intestinal bacteria can produce a dehydroxylated form of RSV, which can also pass through enterocytes and be metabolized into RSV‐glucuronides and RSV‐sulfates.^30^ RSV is mainly excreted by the urinary system.^31^, ^32^ Five metabolites of RSV have been detected in human urine: RSV monosulfate; two isomeric forms of RSV monoglucuronide; monosulfate dihydroresveratrol; and monoglucuronide dihydroresveratrol.^14^ Rapid absorption, poor bioavailability, and low aqueous solubility are some of the crucial limitations of the clinical application of RSV. Therefore, novel methodological approaches, such as nanoparticles and nanobubbles, have been used to improve the poor aqueous solubility and the low bioavailability of RSV.^33^, ^34^, ^35^, ^36^

The toxicity of RSV mainly depends on its dosage. At single dose of <1 g RSV showed no obvious side effects.^37^, ^38^ Upon continuous administration of RSV at 2.5 g/day for a month, side effects such as nausea, vomiting, diarrhea, and liver dysfunction may appear.^39^ Pollack *et al*.^40^ reported frequent diarrhea upon treatment with 2 g RSV twice daily. When the dose was reduced to 1 g twice daily there were no further gastrointestinal side effects. Overall, RSV is generally considered to be safe at dose of ≤1 g/day.

## Molecular Actions of RSV in the Prevention of IDD

**Fig. 1:**
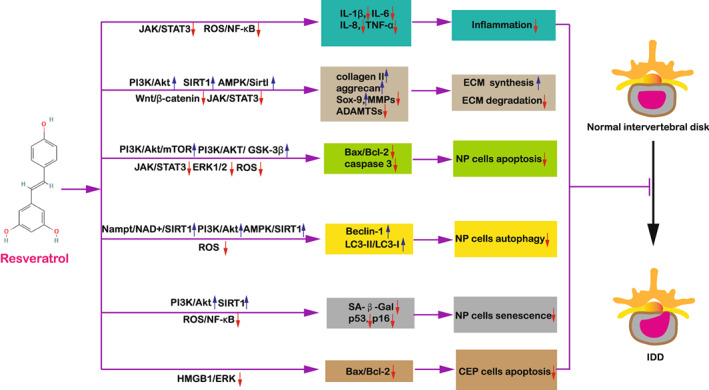
Effects of RSV on IDD. RSV treatment leads to up‐regulation of ECM biosynthesis, down‐regulation of ECM degradation, inhibition of NP cells apoptosis, activation of NP cells autophagy, and inhibition of NP cells senescence, thus attenuating IDD progression

The intervertebral disc acts as the load‐bearing component of the spine and is composed of three closely connected parts: inner NP, peripheral annulus fibrosus (AF) and outer cartilage endplate (CEP).^41^, ^42^, ^43^ IDD is a complex disease including multiple pathological processes. The pathogenesis of IDD mainly involves degradation of ECM, NP cells senescence, apoptosis, autophagy, inflammatory responses and oxidative stress.^44^ NP cells senescence, apoptosis, inflammation and oxidative stress result in the promotion of the ECM degradation.^10^, ^45^, ^46^ What is more, activation of autophagy can also influence the activity of NP cells, thereby regulating ECM homeostasis.^47^ According to the literatures, the RSV showed protective effects on IDD through a variety of mechanisms (Table 1, Fig. 1).

**TABLE 1:** Summary of the *in vitro* researches about pharmacological activities of RSV in IDD

Type of model	Dose	Findings	Molecular mechanism	Reference
Human NP cells	10, 20, 50 μM	HSP90↑, N‐cadherin↑, aggrecan↑, collagen II↑, collagen X↓, IL‐6↓, p‐JAK1↓, p‐STAT3↓	Promoted ECM synthesis and inhibited NP cells apoptosis by blocking IL‐6/JAK/STAT3 pathway	^54^
SD rats NP cells	100 μM RSV	caspase‐3↓, Bcl‐2↑, Bax↓, cleaved PARP↓, p‐Akt/Akt↑	Inhibited IL‐1β‐induced NP cells apoptosis through activation of PI3K/Akt pathway	^21^
SD rats NP cells	100 μM RSV	ROS↓, SA‐β‐Gal activity↓, telomerase activity↑, p16↓, p53↓, MMP‐3↓, MMP‐13↓, ADAMTS4↓, collagen II↑, aggrecan↑	Inhibited NP cells senescence in inflammatory environment	^55^
Pig NP tissues	50 and 100 μM	GAG↑, HYP↑, aggrecan↑, collagen II↑, p‐Akt/Akt↑, SOX‐9↑	Promoted ECM biosynthesis through activating the PI3K/Akt signaling pathway under mechanical compression	^69^
Human NP Cells	50 μM RSV	SIRT1↑, aggrecan↑, collagen II ↑, MMP‐1↑	Promoted ECM biosynthesis by activating the SIRT1 expression	^73^
Human NP Cells	25 μM	SIRT1↑, collagen II ↑, MMP‐13↓, ADAMTS‐5↓, p21↓, p16↓	Promoted ECM biosynthesis, suppressed cellular senescence, increased cell proliferation, and restrained the apoptosis by activating the SIRT1 expression	^72^
Human NP Cells	100 μM	aggrecan↑, collagen II↑, SIRT1↑, β‐catenin↓	Promoted ECM biosynthesis by activating the SIRT1 expression *via* Wnt/β‐catenin signaling pathway	^22^
SD rats NP cells	1 μM E2, 10 μM, 100 μM and 200 μM RSV	collagen II↑, aggrecan↑, MMP‐3↓, MMP‐13↓, p‐Akt/Akt↑, caspase‐3↓	Combined with E2 inhibited NP cells apoptosis and ECM degradation by activating PI3k/Akt pathway	^74^
Bovine NP cells	10, 50, 200 μM RSV	MMP‐13↓, ADAMTS4↓, PG↑, CREB↓	Slowed the progression of IDD by inhibiting catabolism and promoting PG synthesis in NP cells	^75^
SD rats NP cells	100 μM RSV	caspase‐3↓, caspase‐9↓, mitochondrial membrane potential↑, ROS↓	Inhibited SNP induced NP cells apoptosis by scavenging ROS	^20^
Pig NP tissues	50 μM, 100 μM RSV	Caspase‐9 activity↓, Caspase‐3 activity↓, Bcl‐2↑, Bax↓, caspase‐3↓, p‐ERK1/2↓	Inhibited mechanical compression induced NP cells apoptosis through the inhibition of ERK1/2 pathway	^79^
SD rats NP cells	100 μM	Bcl‐2↑, Bax↓, caspase‐3↓, p16↓, p53↓, p‐Akt/Akt↑	Inhibited high glucose induced NP cells apoptosis and senescence by activating the PI3K/Akt pathway	^82^
Human NP cells	100 μM RSV 1 mM E2	caspase‐3↓, p‐mTOR/mTOR↑, P‐GSK‐3b/GSK‐3b↑, NF‐κB↓	Combined with E2 prevented IL‐1β induced NP cells apoptosis *via* the PI3K/AKT/mTOR and PI3K/AKT/ GSK‐3β Pathway	^84^
Human NP cells	50 μM	mitochondrial membrane potential↑, ROS↓, LC3II/LC3 I↑, p62↓	Attenuated oxidative stress induced mitochondrial dysfunction by activation of autophagy	^87^
SD rats NP cells	50 μM	aggrecan↑, collagen II↑, LC3II/LC3 I↑, beclin‐1↑, GAG↑, p‐Akt/Akt↑	Promoted ECM biosynthesis through activating autophagy *via* the PI3K/Akt pathway under oxidative damage	^23^
Human NP cells	24 μM RSV	MMP‐3↓, SIRT1↑, p‐AMPK↑, beclin‐1↑, LC3II/LC3 I↑	Inhibited TNF‐a–induced MMP‐3 expression by activating autophagy *via* SIRT1/AMPK pathway	^88^
Human NP cells	8 μM RSV	LC3II/LC3 I↑, Nampt↑, NAD + ↑, SIRT1↑	Promoted autophagy of NP cells by activating Nampt/NAD+/SIRT1 signaling pathway	^89^
SD rats NP cells	30 μM, 60 μM RSV	SA‐β‐Gal activity↓, ROS↓, telomerase activity↑, p16↓, p53↓	Inhibited NP cells senescence induced by mechanical overload through inhibition of ROS/NF‐κB pathway	^19^
SD rats CEP cells	10 μM, 20 μM, 30 μM, 40 μM RSV	TNF‐α↓, IL‐10↑, HMGB1↓, p‐ERK↓, Bax ↓, Bcl‐2↑	Inhibited CEP cells apoptosis by suppressing HMGB1/ERK signaling pathway	^95^

### Anti‐Inflammation Effects

Inflammatory response directly participates in the progression of IDD. It can also lead to secondary low back pain and radicular symptoms.^48^ The expression of pro‐inflammatory cytokines, such as TNF‐α, IL‐1β and IL‐6 are found to be increased in the intervertebral disc tissues of IDD patients.^49^, ^50^, ^51^ They have been shown to involve in various disease processes of IDD, including ECM degradation, cell apoptosis and senescence.^52^, ^53^ Wu *et al*.^54^ revealed that RSV could increase the expression of heat shock protein 90 (HSP90) and N‐Cadherin in NP cells and promoted ECM biosynthesis by blocking IL‐6‐JAK/ STAT3 positive feedback loop. Jiang *et al*.^21^ found that RSV exhibited its anti‐inflammatory effects on IL‐1β‐mediated NP cells *via* activating PI3K/Akt signaling pathway, thereby attenuating inflammation induced apoptosis of NP cells. Li *et al*.^55^ discovered that RSV could inhibit NP cells senescence in IL‐1β and TNF‐α induced inflammation environment. Wuertz *et al*.^56^ reported that the administration of RSV significantly inhibited the expression of IL‐6 and IL‐8 in the human NP. In another study, RSV could reduce TNF‐a and IL‐1 levels in radiculopathic pain model rats, leading to improved pain behavior in animal models.^57^ Taken together, RSV can inhibit the production of pro‐inflammatory cytokines such as IL‐1β, IL‐6, IL‐8 and TNF‐α, and thus ameliorate IDD progression mediated by inflammation.

### Induction of the ECM Metabolic Homoeostasis of NP Cells

The NP is a hydrated gel‐like tissue, which is the main functional structure of intervertebral disc and can be adjusted according to mechanical stress stimuli.^58^, ^59^, ^60^ Studies have reported that the preservation of normal intervertebral disc mechanical function is dependent on the ECM homeostasis, and reduced biosynthesis and increased degradation of the ECM during IDD leads to mechanical dysfunction of the disc and aggravates disc degeneration.^61^, ^62^, ^63^ NP cells (the main cell type in NP) are responsible for the biosynthesis and maintenance of the ECM. Matrix metalloproteinases (MMPs) and a disintegrin and metalloproteinases with thrombo‐spondin motif proteins (ADAMTSs) are the main force of ECM remolding during IDD.^64^, ^65^ At the cellular level, mechanical load, oxidative stress, high glucose and inflammatory response can induce ECM catabolism.^65^, ^66^, ^67^, ^68^ Han *et al*.^69^ investigated the effects of RSV on ECM under mechanical compression. The results showed high‐magnitude mechanical compression seriously decreased ECM content, while treatment with RSV could increase the proteoglycan content, biochemical content (glycosaminoglycan (GAG) and hydroxyproline (HYP)) and anabolic factors (Sox‐9, aggrecan and collagen II) of ECM. Further research indicated that RSV reversed mechanical load induced ECM degradation by activating the PI3K/Akt pathway in a dose‐dependent manner.^69^ Previous studies showed that activating Sirtuin 1(SIRT1), a NAD (+)‐dependent deacetylase, could also promote ECM biosynthesis.^70^, ^71^, ^72^ Wu *et al*.^73^ compared the expression of SIRT1 and ECM biosynthesis‐related factors in the NP tissues removed from patients with IDD and lumbar fracture (control group). The results of immunohistochemistry and quantitative fluorescence PCR showed that the expressions of SIRT1, collagen II and aggrecan in IDD patients were lower, while the expression of MMP‐1 was higher than those in control patients. However, after RSV treatment, the above results were reversed. Therefore, they suggested that RSV promotes ECM expression by altering MMP‐1 and SIRT1 expression, thereby reversing IDD progression.^73^ Similarly, Guo *et al*.^72^ extracted NP cells from elderly patients with lumbar disc herniation and treated the degenerative NP cells with RSV. The results showed that RSV could down‐regulate catabolic facros including MMP‐3 and MMP‐13 as well as up‐regulate the anabolic factors including collagen II and aggrecan by activating SIRT1.Another study showed that RSV activated SIRT1 through inhibiting Wnt/β‐catenin signaling pathway, and thus ameliorated degradation of the ECM and promoted ECM biosynthesis.^22^ Inflammatory response is another important factor leading to ECM degradation. In a study by Yang *et al*.,^74^ combined use of 17β‐estradiol (E2) and RSV could reverse the down‐regulation of collagen II and aggrecan induced by IL‐1β in rat NP cells *via* PI3K/Akt/caspase‐3 pathway. Wu *et al*.^54^ found that RSV promoted the expression of collagen II and aggrecan by suppressing JAK/STAT3 phosphorylation and decreasing IL‐6 production. In addition, Li *et al*.^75^ found RSV can effectively suppress the IL‐1β induced up‐regulation of catabolic factors including MMP‐1, MMP‐3, MMP‐13, and ADAMTS‐4 and enhance anabolism of proteoglycan. These studies demonstrated that RSV could improve ECM homeostasis under inflammatory environment.^54^, ^74^, ^75^

### Inhibition of NP Cells Apoptosis

The occurrence of IDD is accompanied by high rates of apoptosis, leading to decreasing cell numbers in NP tissue, and therefore disturb the homeostasis of ECM.^76^, ^77^ Hence, inhibiting the apoptosis of NP cells can be an effective approach for IDD treatment.^78^ Oxidative stress accelerates IDD by influencing NP cells apoptosis, autophagy, senescence and DNA methylation.^11^ Li *et al*.^20^ induced an oxidative stress injury model of NP cells using sodium nitroprusside (SNP). The results showed that RSV could reverse the promotion of NP cells apoptosis and overproduction of reactive oxygen species (ROS) and nitric oxide (NO). Actin‐Tracker Green and Tubulin‐Tracker red staining showed RSV could protect NP cells from disruption of cytoskeletal and morphological structure induced by SNP. Notably, its effect on ROS scavenging is comparable to another common ROS scavenger n‐acetyl cysteine (NAC). Zhang *et al*.^79^ identified a significant increase in apoptosis ratio of NP cells under mechanical compression in a dose‐dependent manner, and further signaling pathway research indicated RSV treatment appeared to be effective in alleviating NP cells apoptosis *via* ERK1/2 pathway. Recent research has confirmed that diabetes is a potential causative factor of IDD.^80^, ^81^ Wang *et al*.^82^ treated high glucose induced human NP cells with RSV and RSV + PI3K inhibitor LY294002, respectively. The results showed that the apoptosis ratio of NP cells treated with RSV was lower than those treated with RSV + LY294002, while the Akt phosphorylation (p‐Akt) level showed the opposite trend. Therefore, they concluded that RSV inhibited high glucose‐induced NP cells apoptosis by activating the PI3K/Akt signaling pathway. Research has also demonstrated that inflammation response is directly related with NP cells apoptosis.^82^ Inhibiting inflammation may be another important way to suppress NP cells apoptosis.^10^, ^83^ Jiang *et al*.^21^ induced NP cells apoptosis using IL‐1β and found that the expression of caspase‐3 decreased in NP cells after RSV treatment but increased after LY294002 treatment, so that RSV attenuated inflammation mediated NP cells apoptosis by activating the PI3K/Akt signaling pathway. In addition, Wu *et al*.^54^ found that RSV inhibited NP cells apoptosis by blocking IL‐6/JAK/STAT3 signaling pathway. Yang *et al*.^74^ found that single application of E2 or RSV both inhibited IL‐1β induced NP cells apoptosis. However, combined use of E2 and RSV reduced the cytotoxic effect of IL‐1β on NP cells more efficiently. Furthermore, Bai *et al*.^84^ discovered that RSV combined with E2 enhanced PI3K and Akt expression, and subsequently promoted the activation of p‐mTOR and p‐GSK‐3b, which contributed to decreased caspase‐3 levels. They concluded that combined use of RSV and E2 attenuated IL‐1β‐induced cell apoptosis and recovered cell viability *via* activating PI3K/AKT/mTOR and PI3K/AKT/GSK‐3β signaling pathways.

### Promotion of NP Cells Autophagy

Autophagy is a self‐protective cellular mechanism that removes damaged or senescent organelles, and considered to be an important cellular metabolic process.^85^, ^86^ The exact role of autophagy in IDD remains controversial. At present, most studies reported that the activation of autophagy could ameliorate IDD progression, while relatively few studies showed that excessive activation of autophagy could accelerate IDD progression.^47^ The impacts of RSV on autophagy were investigated in different studies. Zhang *et al*.^87^ discovered that H_2_O_2_ enhanced intracellular ROS expression and induced mitochondrial dysfunction in human NP cells, which was characterized by down‐regulation of ATP and mitochondrial membrane potential levels. However, RSV treatment promoted autophagic flux as well as exerting protective effects on mitochondrial dysfunction and cell apoptosis induced by H_2_O_2_. Therefore, they concluded that RSV attenuated oxidative stress induced mitochondrial dysfunction by activation of autophagy.^87^ In another study, Gao *et al*.^23^ found RSV promoted ECM biosynthesis through stimulating autophagy *via* the PI3K/Akt pathway under oxidative damage. Wang *et al*.^88^ discovered that RSV activated autophagy through up‐regulating SIRT1 expression *via* stimulating upstream regulator AMPK in TNF‐a treated human NP cells and thereby inhibited the expression of MMP‐3. They concluded that RSV attenuated the catabolic effect through down‐regulating TNF‐a induced MMP‐3 expression *via* stimulating autophagy mediated by the AMPK/SIRT1 signaling pathway. In addition, Shi *et al*.^89^ found that RSV can promote autophagy of NP cells by activating Nampt/NAD+/SIRT1signaling pathway and thus accelerating the progression of IDD.

### Inhibition of NP Cells Senescence

NP cells senescence is a common feature during IDD progression and is often demonstrated to be positively correlated with IDD grade.^90^, ^91^ Age‐related β‐galactosidase (SA‐β‐Gal) is considered as a reliable marker of cellular senescence.^92^, ^93^ Recent research has shown that RSV attuned cellular senescence by down‐regulation of SA‐β‐Gal activity, inhibits G0/1 cell cycle arrest, and production of senescent markers (p53 and p16) and up‐regulation of telomerase activity.^19^, ^55^, ^82^ For further mechanism research, Jiang *et al*.^19^ discovered that RSV alleviated NP cell senescence by inhibiting ROS generation and activity of the NF‐κB under mechanical compression in a dose‐dependent manner. Wang *et al*.^82^ found that RSV activated the PI3K/Akt pathway by reducing ROS production, and thereby inhibiting high glucose‐induced cellular senescence. Li *et al*.^55^ demonstrated that RSV reversed the increase of NP cell senescence and promoted ECM biosynthesis in TNF‐α and IL‐1β induced inflammatory environment. Additionally, Guo *et al*.^72^ found that the RSV suppressed cellular senescence and attenuated the apoptosis of NP cells through activating SIRT1.

### Inhibition of CEP Cells Apoptosis

The CEP plays an important role in maintaining the normal shape of the vertebral body, biomechanical stabilization and solute transportation. The calcification or cell apoptosis of CEP hinders nutrient supplement, oxygen transmission and excretion of metabolic wastes from NP, and in depth study of the mechanism of CEP degeneration can provide a novel idea for the prevention and treatment of IDD.^94^ Presently, there is only one study investigating the effects of RSV on CEP. Hu *et al*.^95^ reported that RSV could inhibit TNF‐ α expression, increase IL‐10 expression, reduce Bax/Bcl‐2 and therefore inhibit CEP cells apoptosis. Gene chip analysis indicated that the regulatory mechanism of RSV on CEP cells apoptosis was mediated by inhibiting HMGB1/ERK signaling pathway.^95^

## Effects of RSV on IDD in Animal Studies

Researchers investigated the effects of RSV on IDD *in vivo* using rodent and rabbit models (Table 2). Kwon^96^ induced IDD by annular puncture of lumbar discs in New Zealand white rabbits. MRI showed that compared to the vehicle (DMSO) group, the RSV treatment group had an increased T2 weighted image signal, and the modified Thompson MRI grade was lower than that of the vehicle group. Examination of IDD related gene expression showed aggrecan expression in the RSV group was higher than that of the vehicle group, while the expression level of MMP‐13 was lower than that of the vehicle group. Hematoxylin–eosin (HE) staining showed RSV could ameliorate the cellular characteristics of IDD caused by annular puncture, such as fibroblast‐like cells and severe fibrosis of extracellular components. In another study by Zhang *et al.*,^87^ Safranine O/fast green staining showed that the OD value of central NP tissue in the RSV injection group was higher than that of the operation group, which indicated the beneficial effects of RSV on ECM biosynthesis. Terminal deoxynucleotidyl transferase dUTP nick end labeling (TUNEL) staining showed that the proportion of NP cells apoptosis in the operation group was higher than that of the sham operation group, while injection of RSV reduced the ratio of apoptotic cells. Transmission electron microscope (TEM) results showed that the mitochondria in the NP cells of the operation group were swollen, indicating that mitochondria were damaged, while the ratio of damaged mitochondrial damage was ameliorated by RSV injection. These results showed that RSV injection reduces the apoptosis of NP cells by reducing mitochondrial damage and therefore effectively prevented the progression of IDD. Xia *et al*.^97^ induced an C57BL/6J mice IDD model using intervertebral disc puncture. MRI examination showed that RSV reduced the modified Thompson MRI grade scores. The immunohistochemical staining also showed that RSV could promote the expression of collagen II and PCNA as well as decrease the number of p16 positive cells. Furthermore, knockout of the SIRT1 gene could reverse the above results. In conclusion, they suggested that SIRT1 plays a protective role in IDD progression, and down‐regulated expression of SIRT1 could lead to NP cells senescence, thereby accelerating IDD progression. RSV also showed preventative role against IDD by promoting cells proliferation and inhibiting the senescence of NP cells through activating SIRT1 and its downstream molecule, p16.

**TABLE 2:** Summary of the *in vivo* researches about pharmacological activities of RSV in IDD

Type of model	Route	Dose	Duration	Findings	Reference
New Zealand rabbits IDD model by percutaneous annular puncture	Injection	15 μL of 100 μM RSV	once every 2 weeks for 4,8 and 16 weeks	modified Thompson MRI grade scores↓, MMP‐13↓, aggrecan↑, histological grades↓	^96^
New Zealand rabbit IDD model by annulus fibrosus puncture method	Injection	50 μL of 50 μM RSV	8 weeks	MRI index↑, ECM synthesis ↑, proportion of NP cells apoptosis ↓, proportion of damaged mitochondria↓	^87^
C57BL/6J mice IDD model by the annulus needle puncture	Oral	100 mg RSV/kg body weight per day	At 1 and 4 weeks after puncture, lasted for 7 days each time	SIRT1↑, modified Thompson MRI grade scores↓, histological grades↓, collagen II↑, p16(+) cells↓, PCNA (+) cells↑	^97^
SD rats lumbar radiculopathy model induced by autologous NP	Injection	0.1 mL of 50 μM RSV	21 days	pain threshold↑, pro‐inflammatory cytokine levels↓	^56^
SD rats lumbar radiculopathy model induced by autologous NP	Injection	0.1 mL of 50 μM RSV	21 days	pain threshold↑, cell edema↓, focal hyperaemia↓, pro‐inflammatory cytokine levels↓	^57^
New Zealand rabbits IDD model by annular puncture	Injection	25 μL of 100 mg/ml RSV/PLGA NBs	8 weeks	disc area↑, MRI index↑, aggrecan↑, SIRT1 (+) cells↑	^36^

Radiculopathic pain is the main symptom of IDD. The von Frey filament test is commonly used to evaluate paw withdrawal threshold, and lower withdrawal thresholds are considered as sign of mechanical hypersensitivity, which is correlated to pain behavior in animal models.^98^, ^99^ Researchers applied autologous NP on the rat dorsal root ganglion (DRG) to conduct IDD pain model. The results showed that compared to the vehicle (saline) group, RSV promoted withdrawal thresholds for up to 14 days after injection, which indicated that RSV could attenuate NP‐mediated pain.^56^, ^57^ Inflammation inhibition are known as potential targets for ameliorating pain in IDD.^100^ Wuerts *et al*.^56^ found that RSV suppressed the expression of IL‐6, IL‐8, MMP1, MMP3, and MMP13 and thereby induced anti‐inflammatory and anti‐catabolic effects. They believed that decreased pro‐inflammatory cytokine levels may be the underlying mechanism of pain reduction.^56^ Lin *et al*.^57^ used HE staining to show that RSV could improve cell structure, with decreased edema and focal hyperaemia, which indicated the inflammatory response was suppressed. Additionally, immunohistochemical staining showed that RSV could reduce TNF‐a and IL‐1 levels caused by neuron surgery.^57^

The application potential of RSV is limited by its poor water solubility, poor stability, fast metabolism, and difficulty in reaching an effective blood concentration in intervertebral discs.^101^ To solve this problem, the target release of RSV by ultrasound (US)‐mediated poly (lactic‐co‐glycolic acid) nanobubbles (NBs) have been investigated by Shen *et al*.^36^ The RSV‐embedded NBs were synthesized using a double‐emulsion method. The active NP cell‐targeting biomarker CDH2 antibody (AbCDH2) was further conjugated to the NBs using a carbodiimide method.^36^ The results showed that the RSV/AbCDH2 NBs had a high loading capacity and drug release efficiency. Furthermore, US can also enhance the release of RSV from RSV/AbCDH2 NBs and increase local blood drug concentration. In a rabbit IDD model, local injection of US mediated RSV/AbCDH2 NBs effectively improved characteristics of IDD progression including MRI index, aggrecan expression, and SIRT1 expression compared with using RSV alone.^36^

At present, rodent trauma models are the main animal models used to investigate the effects of RSV on IDD, which do not reflect the biomechanical characteristics of the natural degeneration of the human body.^102^ The recent mouse standing model may provide a better direction for related researches.^103^, ^104^ For instance, Lao *et al*.^103^ found that the movement pattern of mice was similar to that of humans when standing and jumping with their lower limbs. The custom‐made hot plate cage was used to provide the axial biomechanical load of the spine. The fissures in AF increased, the height of intervertebral disc and CEP decreased, and the metabolic homeostasis of ECM was disturbed with the accumulation of biomechanical loading and time, forming an IDD model. What's more, notochordal cells are retained in rodent NP tissues, which disappear in human adults. At present, no suitable molecules have been found to distinguish notochord cells in NP tissues.^102^, ^105^ Therefore, the existence of notochord cells may affect the research results. In order to eliminate the gap between *in vivo* models and humans, a more clinically relevant animal model should be developed to better mimic the complexity of the human intervertebral disc and IDD progression.

### Conclusion and Perspective

In conclusion, RSV shows substantial protective roles in the progression IDD. Mechanism researches reveal that RSV could effectively inhibit the apoptosis and senescence as well as promote autophagy of NP cells. It also exerts anabolic and anti‐catabolic effects on ECM, which are crucial for the regeneration of damaged intervertebral disc. Multiple signaling pathways, such as PI3K/Akt, NF‐κB, AMPK/SIRT1, and ERK1/2, are the common target signaling pathways of RSV in IDD treatment. What is more, inflammatory and oxidative stress could also be suppressed by RSV treatment. However, the published studies are limited to preclinical studies, and evidence of RSV‐containing drugs for the therapy of IDD has yet to be investigated in clinical trials to confirm the preliminary results obtained from previous researches. And the current studies are mainly focused on NP, and the effects of RSV on AP and CEP also need systematic investigation. In addition, more studies should be performed to verify the synergistic effects of RSV combined with traditional drugs in the treatment of IDD in order to enhance efficacy and decrease the drug resistance and side effects. Moreover, the poor aqueous solubility and rapid metabolism of RSV might restrict its clinical application. As previously reported, RSV released by US‐mediated RSV/AbCDH2 NBs is a promising strategy to enhance its bioavailability and pharmacological efficacy in IDD treatment. With this consideration, mechanisms to promote the absorption of RSV are also worthy of investigation.

## Author Contributions

The concept of the manuscript was devised by Yan‐zheng Gao. Ming‐yang Liu and Kai‐guang Zhang performed the overall literature searches. Ming‐yang Liu and Liang Zhang were in charge of writing. Tables and Figure were designed by Ming‐yang Liu. Hai‐jun Li, Wei‐dong Zang and Yan‐zheng Gao discussed the content of the article and gave suggestions.
